# Prediction tools for the personalized management of soft-tissue sarcomas of the extremity

**DOI:** 10.1302/0301-620X.104B9.BJJ-2022-0647

**Published:** 2022-09-01

**Authors:** Ibtissam Acem, Michiel A. J. van de Sande

**Affiliations:** 1 Department of Surgical Oncology and Gastrointestinal Surgery, Erasmus MC Cancer Institute, Rotterdam, the Netherlands; 2 Department of Orthopaedic Oncology, Leiden University Medical Centre, Leiden, the Netherlands

**Keywords:** Extremities, Soft-tissue sarcoma, Adjuvant treatment, Nomogram, Predictions, Model performance, Decision curve analysis, Personalized care, Soft-tissue sarcomas, extremities, prognosis, clinicians, oncology, sarcomas, (Neo)adjuvant chemotherapy, chemotherapy, Physicians, radiotherapy

## Abstract

Prediction tools are instruments which are commonly used to estimate the prognosis in oncology and facilitate clinical decision-making in a more personalized manner. Their popularity is shown by the increasing numbers of prediction tools, which have been described in the medical literature. Many of these tools have been shown to be useful in the field of soft-tissue sarcoma of the extremities (eSTS). In this annotation, we aim to provide an overview of the available prediction tools for eSTS, provide an approach for clinicians to evaluate the performance and usefulness of the available tools for their own patients, and discuss their possible applications in the management of patients with an eSTS.

Cite this article: *Bone Joint J* 2022;104-B(9):1011–1016.

## Introduction

Soft-tissue sarcomas represent a group of rare and heterogeneous malignant neoplasms, with more than 100 histological subtypes.^1^ They arise from mesenchymal cells and account for 1% of adult malignancies.^2^ The estimated incidence is 4.71 per 100,000 people per year in Europe.^3^ They may occur in any anatomical site, but the limbs are the most common primary site for a soft-tissue sarcoma.^4,5^ Because of the heterogeneity in presentation and outcome within the spectrum of soft-tissue sarcomas of the extremities (eSTSs), several prognostic instruments have been developed to classify patients with these tumours into risk groups to optimize their management. Historically, conventional staging systems such as the American Joint Committee on Cancer TNM classification were widely used for the stratification of patients.^6^ However, important prognostic patient and tumour-related factors such as age and histological subtype are not incorporated in the TNM staging system. In recent years, several new prognostic instruments such as prediction tools and nomograms have been developed for eSTSs. In general, these tools are easier to use through applications on smartphones, are more accurate (as they generate an individual prognosis based on multiple characteristics that may vary simultaneously), and provide a prognosis which is more easily understood when compared with conventional staging systems. In this annotation, we discuss the current concepts of managing eSTSs, explore the available prediction tools for the management of these rare tumours, provide clinicians and researchers instruments to assess which tool to use, and discuss the current and future applications of prediction tools for clinical decision-making and the personalized management in eSTSs.

Several clinical guidelines have been developed for the management of eSTSs.^7,8^ The treatment should occur in a multidisciplinary team using a multimodal approach. Several studies have shown that the treatment of a STS in high-volume centres is associated with better oncological outcomes.^5,9-11^ This underlines the importance of centralization of sarcoma care in centres with a dedicated sarcoma team.

Surgery with complete surgical margins is the standard treatment for a localized eSTS. (Neo)adjuvant radiotherapy is typically indicated in high-grade eSTSs with a high risk of local recurrence or of incomplete surgical margins. The most important factors influencing the recommendation for radiotherapy are the anticipated surgical margin, the grade, size, and location of the tumour, and its histological subtype.^12^ It has been shown that a marginal resection after radiotherapy may not compromise local control or overall survival.^13,14^ Also, recent studies suggest that after a R1 excision^6^ (with microscopically evident residual tumour) or unplanned excision, further excision may be postponed after multidisciplinary discussion until a local recurrence occurs, without compromising the overall survival or distant control.^15,16^ However, the clinical guidelines recommend systematic re-excision in patients with an incomplete surgical margin if R0 re-resection (negative surgical margins) is feasible.^6-8^

There is no clear preference about the timing of radiotherapy. Local control and overall survival are comparable after both neoadjuvant and adjuvant therapy.^17-20^ Traditionally, radiotherapy was often offered postoperatively, as short-term wound complications are less common after adjuvant therapy. However, neoadjuvant therapy results in less long-term morbidity such as fibrosis, oedema, and joint stiffness compared with adjuvant therapy.^17-20^ Given that the short-term complications are manageable in specialized sarcoma centres, radiotherapy is nowadays typically offered preoperatively.^7,21^

(Neo)adjuvant chemotherapy may be indicated in patients with a high risk of developing distant metastasis or of dying. Perioperative chemotherapy is not routine treatment in the management of a primary eSTS, but may be offered in a selected group of high-risk patients after multidisciplinary discussion. The chemosensitivity of the histological subtype should be taken into consideration.

Despite several randomized and non-randomized studies on the added value of perioperative chemotherapy in the management of an eSTS, its role is still widely debated.^22-34^ To date, five randomized trials comparing anthracycline and ifosfamide-based (neo)adjuvant chemotherapy in addition to routine treatment versus routine treatment alone have been performed.^22-26^ None of these found a survival benefit in the chemotherapy arm of the trial. However, most trials included low-risk patients with low-grade tumours and small superficial tumours. Three of the five trials were also closed prematurely because of poor patient recruitment.^23,25,26^

Recent studies have shown improved survival for patients treated with anthracycline- and ifosfamide-based chemotherapy in localized eSTSs in high-risk patients.^33-35^ These patients were identified using prediction tools, which anticipate individual risks of metastasis formation and death based on characteristics of the patient, tumour, and treatment.^36,37^ A survey among sarcoma specialists reported that 81% consider the use of a prediction tool for the indication of (neo)adjuvant chemotherapy in patients with a primary eSTS,^12^ marking a recent trend to a more patient-tailored approach in the management of these tumours.

Treatment with (neo)adjuvant isolated limb perfusion with tumour necrosis factor-alpha plus melphalan and (neo)adjuvant regional hyperthermia, combined with chemotherapy, may also be an option for limb-preserving treatment after multidisciplinary discussion in specialist centres.^7,8,38^

Prediction tools in the form of a nomogram or a computer- or smartphone-based calculator are commonly used to estimate oncological events such as the risk of recurrence and death.^12,39^ These tools generate individual probabilities of an event based on a combination of factors accounting for the fact that patients have many characteristics that may vary simultaneously. This results in the identification of a more accurate individual prognosis, which is easier to explain compared with conventional staging systems in cancer. Prediction tools allow decisions to be made about treatment in a more patient-tailored manner. The last decade has seen an enormous increase in the development and publication of prognostic tools in medicine, and the development of several of these tools in the field of the management of eSTSs.^36,37,40-48^

An overview of published prediction tools for patients with a primary STS is shown in Table I.^36,37,40-54^ Diagnostic models and histologically-specific models are not included. All prediction tools have different inclusion criteria. Three tools included only STSs of the extremities, while others included sarcomas in other sites.^36,37,43^ Some studies included patients with metastatic disease or local recurrence at the time of presentation.^42,45-48^ One study combined bone and soft-tissue tumours in the prediction tool.^47^ All tools included sarcoma-specific survival or overall survival as an outcome of the model,^36,37,40,42,45-48^ except for the nomogram of Cahlon et al.^43^ Only four studies were externally validated.^36,37,40,42^

**Table I.** Overview of published prognostic tools for soft-tissue sarcoma (excluding histology-specific tools).

Study (name)	Population	Primary endpoint	Predictors	Dynamic predictions	Validation
Kattan et al 2002;^40^ Mariani et al 2005^41^ (MSKSN)	Aged > 16 yrs with primary, non-metastatic, STS treated with surgery	12-yr SSS	Age, size,* grade, histological subtype, depth, site	No	External^49,50^
Sampo et al 2012^42^	Aged > 16 yrs with non-metastatic primary or locally recurrent eSTS or trunk wall STS	10-yr SSS	Size,* grade, depth, site, necrosis, vascular invasion	No	External^42^
Cahlon et al 2012^43^	Aged > 16 yrs with primary, non-metastatic, eSTS treated with limb-sparing surgery alone (excluding perioperative RTX and CTX)	3-, 5-yr LR	Age,* size,* grade, histological subtype, margin	No	Internal^43^
Callegaro et al 2016^37^ (Sarculator)	Aged > 18 yrs with primary (non-recurrent and non-metastatic) eSTS operated with curative intent	5-,10-yr OS; 5-, 10-yr DM	Age, size, grade, histological subtype	Yes^51^	External^37,50-52^
Van Praag et al 2017;^36^ Smolle et al 2019^44^ (PERSARC)	Aged > 18 yrs with high-grade, primary (non-recurrent and non-metastatic) eSTS operated with curative intent	3-, 5-, 10-yr OS; 3-, 5-, 10-yr DM; 3-, 5-, 10-yr LR	Age, size, grade, histological subtype, depth, margin, RTX	Yes^53^	External^44,54^
Sekimizu et al 2019^45^	Aged > 18 yrs with primary (N0M0 or N1M0), eSTS and trunk STS operated with curative intent	2-yr LR; 2-yr DM; 2-yr OS	Age,* size, grade, histological subtype, depth, site, margin, sex, nodal metastasis	No	Internal^45^
Zhang et al 2019^46^	Aged > 18 yrs with primary STS surgically treated	3-, 5-yr OS; 3-, 5-yr SSS	Age,* size,* grade, histological subtype, sex, stage,† marital status, insurance status	No	Internal^46^ ‡
Xu et al 2020^47^	Patients with bone and soft-tissue tumours (except from the heart)	3-mth OS; 3-mth SSS; 3-mth non-SSS	Age (cat), grade, site, surgery, sex, stage,† T-stage, brain metastasis, lung metastasis, laterality, race	No	No
Tu et al 2021^48^	Patients with primary STS	1-, 2-, 3-yr OS	Age,* size,* grade, histological subtype, surgery, RTX, CTX, lung metastasis	No	Internal^48^ ‡

* Recorded as categorical.

† Stage includes localized, regional, or distant disease.

‡ Stated in the paper as external validation; however, the validation cohort was a random split from the same source population (training and validation cohort both from the Surveillance, Epidemiology, and End Results dataset), which is considered to be internal validation.^55^

CTX, chemotherapy; DM, distant metastasis rate; eSTS, soft-tissue sarcoma of the extremity; LR, local recurrence rate; MSKSN, Memorial Sloan Kettering Sarcoma Nomogram; OS, overall survival; PERSARC, PERsonalised SARcoma Care; RTX, radiotherapy; SSS, sarcoma-specific survival; STS, soft-tissue sarcoma.

Two prediction tools, Sarculator and PERsonalised SARcoma Care (PERSARC), included dynamic predictions.^51,53^ Both dynamic tools were externally validated.^51,54^ These tools usually predict oncological outcomes at a certain timepoint (e.g. five-year overall survival) at the time of surgery. However, the prognosis of a patient may change with the passage of time. For example, the longer the patient is disease-free after surgery, the lower the chance of recurrence and the better the prognosis, and those who develop a recurrence during follow-up will have a worse prognosis compared with those who do not. Dynamic predictions take these time-varying variables into account, and can predict the prognosis at various times during follow-up.

All prediction tools in eSTS include patient- and tumour-specific characteristics. Five of nine studies also included treatment-related variables in their nomogram.^36,43,45,47,48^ Besides these clinical predictors, the prognostic ability of other factors such as gene expression profiles, radiomics, transcriptomics, proteomics, and other multiomics have been widely investigated.^56-62^ However, the assessment of the added value of these promising predictors and models, compared with the existing tools, and further external validation, are required.

After a careful model-building process, an assessment of how good the predictions of a model are needs to be undertaken. A model’s performance is often expressed in discrimination and calibration.

### Discrimination

Discrimination relates to how well the model could distinguish between patients who experienced an event and those who did not. It is measured by the area under the curve (AUC) of a received operating curve (ROC), also known as the concordance index, Harrell’s c-index, or c-index. The ROC curve is a graph of the sensitivity (true positive rate) against the specificity (false-positive rate) for different cut-off values of the probability of an outcome. The Harrell’s c-index for models of survival is the probability that for all possible pairs of patients, the one with a shorter time-to-event has a higher predicted risk of the event compared with the one with a longer time-to-event. A c-index of 0.5 corresponds to a model that is no better than chance, and a c-index of 1 corresponds to perfect discrimination (the model could perfectly distinguish those with a shorter time-to-event from those with a longer time-to-event).

### Calibration

Calibration estimates how close the predicted risk based on the tool is to the observed risk in the study population. It can be assessed visually in a graph in which the observed probability is plotted against the predicted probability. The 45° line in this graph indicates perfect calibration (the predicted and observed probability are equal). For survival data, this graph is often reported for several clinically relevant timepoints.

Neither discrimination nor calibration are intrinsic properties of a model. These measurements evaluate how well the model performs in a particular cohort. A good discriminative ability is important for the stratification of risk and to identify a high-risk subgroup, while a good calibration is important for informing patients about their prognosis and clinical decision-making.

### Internal versus external validation

The best assessment of the performance of a model is by external validation. Validation is the process of assessing the performance on different populations and the applicability (generalizability) to these populations. Most prediction tools in eSTS only underwent internal validation,^43,45,46,48^ which assesses validity for the institution in which the training or development was undertaken. It assesses the reproducibility of the model in the same underlying population. External validation assesses the validity in a fully independent cohort. Steyerberg^55^ provides a practical approach for, and further explanation of, different techniques of internal and external validation. Poor external validation may often be explained by inadequate development of the model, overfitting due to a relatively small sample size with many candidate predictors, or a single-centre development cohort.

Poor external validation may also be related to true differences between the cohorts used for development and validation. Prediction tools should be updated for new settings (e.g. at different times). This can be done by recalibration, re-estimation of regression coefficients, or by extension of the model with the inclusion of new predictors. For example, one may argue that the accuracy of the predictions of a generic eSTS model in a patient with a malignant peripheral nerve sheath tumour (MPNST) of the extremity, would be less than one based on a MPNST-specific prediction tool in which important MPNST-specific predictors, such as the presence of neurofibromatosis type 1 and rhabdomyoblastic differentiation (triton tumour), are incorporated.^63^ A recent study showed that the discriminative ability of the Sarculator is less in MPNSTs compared with other histological subtypes, such as leiomyosarcomas (c-index: 0.66 vs 0.75, respectively).^52^ This could be a reason to update the Sarculator in patients with MPNST with additional important MPNST-specific predictors. For the extension of prediction tools, a trade-off between the value of prediction and usability or availability to assess the new predictor in clinical practice should be made. Several approaches for updating existing prediction models are described by Steyerberg.^55^

### The use of a prediction tool for personalized care

Formerly, patients with a deep-seated, high-grade tumour with a diameter of > 5 cm were considered high-risk patients.^64^ However, the updated European Society for Medical Oncology guidelines of 2021 no longer use this definition for high-risk patients, stating that prognostic tools, such as Sarculator and PERSARC, could be used to identify high-risk patients, for example, for the indications for the use of (neo)adjuvant chemotherapy.^7^ Both prognostic tools are available as applications that can be downloaded in the Apple App Store and Google Play Store.

Given the variety in eligibility criteria and the differences in the patients included in the development and validation cohorts, it is difficult to compare the performance of the prediction tools based on their reported discriminative ability and other measures of the performance of a model. For the choice of which prediction tool to use in a clinical setting, one should assess whether the populations used for the development and external validation are comparable to one’s own patient population. Furthermore, the outcome of interest, and relevance and availability of the prognostic covariates which are used in the model, should guide the choice for tool.

Besides the applicability of the prediction tool in the physician’s own patients and the corresponding outcomes, the clinical usefulness should be assessed. This can be done using decision curve analysis, in which the net benefit of a prediction tool-assisted decision at different threshold probabilities is identified, and compared with the default decision of an intervention for all patients and one for no patients. The net benefit is defined as the fraction of true positives subtracted from the fraction of false positives at a certain threshold probability, weighted by the relative harm of a false positive and a false negative result.^65^ This weight corresponds to the harm (false positive) to benefit (false negative) ratio.^66^ For example, if we accept four false positives for one true positive, this would correspond to a threshold probability of 20% and a harm to benefit-ratio of 4, which means that missing a true positive is four times worse than having a false positive.

**Fig. 1:**
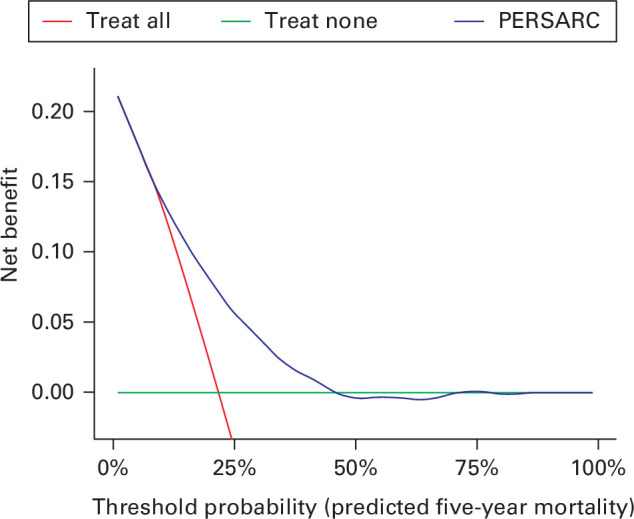
Clinical decision curve plotting net benefit against threshold probability for the PERsonalised SARcoma Care (PERSARC) prediction tool.

In Figure 1, the decision curve analysis of the PERSARC prediction tool is shown in a multicentre cohort of patients with a high grade eSTS, as reported by Acem et al.^33^ As previously described, most sarcoma specialists would consider the use of a prediction tool for the indication for using (neo)adjuvant chemotherapy.^12^ This decision curve analysis illustrates that the PERSARC tool would be clinically useful for the indication for the use of (neo)adjuvant chemotherapy if physicians treat patients with eSTSs with a predicted five-year mortality of between 6% and 45%. The threshold probability refers to the preference of a physician, and reflects how physicians value different outcomes for their patients. If a physician is willing to offer (neo)adjuvant chemotherapy for patients with a predicted five-year mortality of < 6% (five-year survival of more than 94%), he/she should treat all patients with (neo)adjuvant chemotherapy and the prediction tool will not be clinically useful. If a physician is willing to treat patients only if they have a predicted five-year mortality of > 45% (five-year survival of < 55%), he/she should not treat any patient with (neo)adjuvant chemotherapy. Again, in this situation the prediction tool will not be clinically useful. If the threshold probability of a physician lies within the range of 6% and 45%, taking the relative harm and benefit of (avoiding) treatment with (neo)adjuvant chemotherapy into account, the PERSARC model is clinically useful. Acem et al^33^ found a survival benefit for (neo)adjuvant anthracycline- and ifosfamide-based chemotherapy in a subgroup of patients with a five-year predicted survival of ≤ 66% (five-year predicted mortality of 34%). This lies within the range of threshold probabilities in which the model is clinically useful.

### Clinical applications

Besides the use of prediction tools for the indication for the use of (neo)adjuvant treatment,^33,34^ they provide an opportunity to tailor counselling and follow-up appointments. They can help physicians inform their patients about their prognosis and guide decision-making. However, there is little information about whether patients understand the issues, as reflected in satisfaction and quality of life (QoL) with the use of prediction tools in the management of an eSTS. The PERSARC research group has, therefore, started a randomized trial to assess whether the use of PERSARC to support decision-making could contribute to a better-informed choice, less conflict, and improved QoL from a patient’s perspective.

Furthermore, dynamic prognostic tools could be useful for tailoring follow-up regimens to the risk of recurrent tumour formation. The PERSARC group recently published a study in which conditional risks for local recurrence and metastases were predicted using flexible parametric competing risk regression models.^44^ However, the optimal risk threshold upon which an individual patient needs to visit the outpatient clinic or undergo imaging should be further evaluated using microsimulation decision modelling for cost-effectiveness.^67^

Finally, prediction tools are very useful in research; for instance, for the analysis of the stratification of risk to assess the heterogeneity of treatment in clinical trials,^68^ and for the selection of patients for randomized trials.^69^

In conclusion, prediction tools are important instruments for clinical decision-making in the modern world, and facilitate a shift from the one-size-fits-all approach to patient-tailored management of eSTSs. These tools have been shown to be valuable for the identification of high-risk patients, who would benefit from (neo)adjuvant anthracycline and ifosfamide-based chemotherapy.^33,34^ The further development of existing tools with other promising predictors, and recalibration and re-estimation for different settings, are needed to establish their use in clinical practice. For the extension of prediction tools, a trade-off between predictive value and the ability to obtain the predictor in clinical practice should be made, balancing precision and usability.

**Take home message**

- Prediction tools facilitate a shift from a one-size-fits-all approach to patient-tailored management of soft-tissue sarcoma of the extremity (eSTS).

- Multiple prediction tools have been developed in eSTS.

- These tools might be useful as a decision-supporting instrument for (neo)adjuvant chemotherapy and tailored surveillance in eSTS.
